# Disruption of sleep architecture in *Prevotella* enterotype of patients with obstructive sleep apnea‐hypopnea syndrome

**DOI:** 10.1002/brb3.1287

**Published:** 2019-04-08

**Authors:** Chih‐Yuan Ko, Ji‐Mim Fan, An‐Ke Hu, Huan‐Zhang Su, Jiao‐Hong Yang, Li‐Mei Huang, Fu‐Rong Yan, Hua‐Ping Zhang, Yi‐Ming Zeng

**Affiliations:** ^1^ Department of Pulmonary and Critical Care Medicine the Second Affiliated Hospital of Fujian Medical University Quanzhou China; ^2^ Respiratory Medicine Center of Fujian Province Quanzhou China; ^3^ Key Laboratory of Fujian Medical University, Fujian Province University Quanzhou China; ^4^ Department of Endocrinology and Metabolism the Second Affiliated Hospital of Fujian Medical University Quanzhou China; ^5^ Center for Molecular Diagnosis and Therapy the Second Affiliated Hospital of Fujian Medical University Quanzhou China

**Keywords:** enterotypes, microbiota, obstructive sleep apnea‐hypopnea syndrome, polysomnography, *Prevotella*

## Abstract

**Introduction:**

Intermittent hypoxia and sleep fragmentation are critical pathophysiological processes involved in obstructive sleep apnea‐hypopnea syndrome (OSAHS). Those manifestations independently affect similar brain regions and contribute to OSAHS‐related comorbidities that are known to be related to the host gut alteration microbiota. We hypothesized that gut microbiota disruption may cross talk the brain function via the microbiota–gut–brain axis. Thus, we aim to survey enterotypes and polysomnographic data of patients with OSAHS.

**Methods:**

Subjects were diagnosed by polysomnography, from whom fecal samples were obtained and analyzed for the microbiome composition by variable regions 3–4 of 16S rRNA pyrosequencing and bioinformatic analyses. We examined the fasting levels of interleukin‐6 and tumor necrosis factor‐alpha of all subjects.

**Results:**

Three enterotypes *Bacteroides*, *Ruminococcus*, and *Prevotella* were identified in patients with OSAHS. Arousal‐related parameters or sleep stages are significantly disrupted in apnea‐hypopnea index (AHI) ≥15 patients with *Prevotella* enterotype; further analysis this enterotype subjects, obstructive, central, and mixed apnea indices, and mean heart rate are also significantly elevated in AHI ≥15 patients. However, blood cytokines levels of all subjects were not significantly different.

**Conclusions:**

This study indicates the possibility of pathophysiological interplay between enterotypes and sleeps structure disruption in sleep apnea through a microbiota–gut–brain axis and offers some new insight toward the pathogenesis of OSAHS.

**Abbreviations**
AHI: apnea‐hypopnea index
BMI: body mass index
BP: blood pressure
CNS: central nervous system
CompSAS: complex sleep apnea syndromes
CPAP: continuous positive airway pressure
CSA: central sleep apnea
IBD: inflammatory bowel disease
IH: intermittent hypoxia
IL‐ 6: interleukin‐6
LPS: lipopolysaccharide
MAD: apnea‐hypopnea duration
MSA: mixed sleep apnea
NREM: non‐rapid eye movement
OSAHS: obstructive sleep apnea‐hypopnea syndrome
PCA: principal component analysis
PSG: polysomnography
REM: rapid eye movement
SAS: sleep apnea syndromes
SF: sleep fragmentation
TNF‐α: tumor necrosis factor alpha

## 1 INTRODUCTION

Intermittent hypoxia (IH) and sleep fragmentation (SF) are the hallmarks of obstructive sleep apnea‐hypopnea syndrome (OSAHS) (Moreno‐Indias et al., 2015, 2016; Poroyko et al., 2016). IH plays a critical pathophysiological role in of OSAHS, often accompanied by reduced oxygen saturation, increased systemic pressure and bloodstream, excessive sympathetic neural activity, impairment of autonomic function, and apnea episodes end with an arousal of the central nervous system (CNS), ultimately result in vascular endothelial dysfunction and multi‐organ morbid consequences. The underlying mechanism involves inflammation and oxidative stress cascades (Gaspar, Álvaro, Moita, & Cavadas, 2017; Lavie, 2015).

Contrastingly, sleep structure disruption is another risk factor for the pathophysiology of OSAHS, causing major end‐organ morbidity independent of IH (Rosenzweig, Williams, & Morrell, 2013, 2014). Repeated arousals disturbing different stages of sleep are the predominant mechanism underlying OSAHS‐induced brain injury wherein results from disruptions of rapid eye movement (REM) and non‐REM (NREM) (Rosenzweig, Williams, & Morrell, 2014). SF promotes obesity and metabolic abnormalities and may be mediated by concurrent alterations of the host gut microbiota and concurrent systemic and adipose tissue inflammatory alterations accompanied by insulin resistance (Farré, Farré, & Gozal, 2018; Poroyko et al., 2016). Reportedly, prolongation of the N1 stage and shortening of REM times were observed in OSAHS‐induced hypertensive patients; prolongation of the N1 sleep stage also causes elevation of fasting blood glucose (Shao et al., 2018). Elevated serum lipopolysaccharide (LPS)‐binding protein levels might prolong the N1 stage and increase SF, which may be related to increased nighttime respiratory events and arousals (Shao et al., 2018). Interestingly, disturbances in sleep continuity are associated with emotional disorders (Palagini, Baglioni, Ciapparelli, Gemignani, & Riemann, 2013); additionally, the disturbance of sleep structure also contributes to mild cognitive decline in OSAHS (Rosenzweig et al., 2014). However, treating OSAHS patients with continuous positive airway pressure (CPAP) has protective effects on neurocognition, postulated that the microbiota could be modulated during CPAP treatment (Xu et al., 2017), implying that the microbiota might participate in the pathophysiological developed mechanism.

Emerging evidence suggests that the gut microbiota plays a crucial role in modulating the risk of several chronic diseases and maintaining intestinal immunity and whole body homeostasis. These effects have important implications for diseases such as obesity, cardiometabolic abnormalities, inflammatory bowel disease (IBD), and mental illness (Singh et al., 2017). Additionally, the gut microbiota alterations manifested in IH and SF mimic in OSAHS animal models (Moreno‐Indias et al., 2015; Poroyko et al., 2016). However, some of the underlying mechanisms of OSAHS‐related comorbidities remain unclear. Enterotype analysis has been proposed as a useful method to understand human gut microbial communities, including *Bacteroides*, *Ruminococcus*, and *Prevotella* enterotypes, irrespective of ethnicity, gender, age, or body mass index (BMI) (Arumugam et al., 2011). Moreover, enterotypes subdivision provides an attractive framework for linking human disease. For example, *Bacteroides* enterotype has been reported to pose an increased risk for IBD (Conlon & Bird, 2015; Costea et al., 2018; Knights et al., 2014).

Notably, characteristics of IH and SF in OSAHS can trigger the inflammatory response, which then alters the intestinal microbial community composition (Moreno‐Indias et al., 2015; Palagini et al., 2013; Poroyko et al., 2016). Conversely, gut microbiomes can also respond to the brain via the microbiota–gut–brain axis, as has been declared in psychiatric disorders (Sherwin, Sandhu, Dinan, & Cryan, 2016). However, this hypothesis has not been verified for OSAHS. Thus, this study investigated the hypothesis that the microbiota–gut–brain axis is associated with the pathogenesis of OSAHS. We examined whether impaired sleep architecture is associated with gut microbiota alteration by investigating sleep parameters of polysomnography (PSG) data and pro‐inflammatory cytokines in various enterotypes of OSAHS subjects.

## 2 MATERIALS AND METHODS

### 2.1 Subjects

In total, 113 subjects were recruited from July 2017 to January 2018, who were clinically suspicious with OSAHS and committed to sleep examination for the first time at sleep laboratory of Department of Pulmonary and Critical Care Medicine of the Second Affiliated Hospital of Fujian Medical University. Subjects with gastrointestinal diseases, infection, unexplained diarrhea, and antibiotics or probiotics used before recruitment around 1 month were excluded in this study. Subjects were examined during a full night of PSG (SOMNOscreen™ plus PSG^+^; SOMNOmedics GmbH, Randersacker, Germany) by technologists from 10 p.m. to 8 a.m. at the sleep laboratory. Fasting blood and fecal samples were collected the following morning. The Institutional Review Board of the Second Affiliated Hospital of Fujian Medical University approved this study (IRB No. 2017–78).

### 2.2 OSAHS evaluation

All the subjects underwent PSG with a computerized polysomnographic system, simultaneously including electrocardiography, electroencephalography, electromyography, and electrooculography. After one night of examination, apnea‐hypopnea index (AHI) was calculated as the total number of episodes of apnea (continuous cessation of airflow for at least 10 s) and hypopnea (reduction in airflow for ≥ 10 s with oxygen desaturation ≥4%) by dividing the total sleep by events. According to the diagnostic criteria, as reported previously studies (Heizati et al., 2017; Shao et al., 2018), AHI < 15 events/h was defined as non‐OSAHS (control group) and AHI ≥ 15 events/h as OSAHS group.

### 2.3 Cytokine analysis

Interleukin (IL)‐6 and tumor necrosis factor alpha (TNF‐α) were assayed by BD Human Enhanced Sensitivity Cytometric Bead Array Kit (BD Biosciences, New Jersey, USA) as described previously (Kao, Ko, Wang, & Liu, 2016). The standard coefficient of determination (*r* ^2^) was greater than 0.995.

### 2.4 Sampling, DNA extraction, and 16S rRNA gene amplification sequencing

Samples were collected and stored in a Microbiome Test Kit (G‐BIO Biotech, Inc., Hangzhou, China). Magnetic bead isolation was carried out to extract genomic DNA using a TIANamp stool DNA kit (TIANGEN Biotech Co., Ltd., Beijing, China), according to the manufacturer's instructions. The concentration of extracted DNA was determined by a Nanodrop ND‐1000 spectrophotometer (Thermo Electron Corporation, USA), and DNA quality was confirmed using 1.0% agarose gel electrophoresis with 0.5 mg/ml ethidium bromide.

Isolated fecal DNA was used as a template to amplify V3 and V4 hypervariable regions of the bacterial 16S ribosomal RNA gene. V3 and V4 regions were PCR‐amplified (forward primer, 5′‐ACTCCTACGGGAGGCAGCAG‐3′; reverse primer, 5′‐GGACTACHVGGGTWTCTAAT‐3′). The 16S target‐specific sequence contained adaptor sequences permitting uniform amplification of a highly complex library ready for downstream next‐generation sequencing on Illumina MiSeq (Illumina, USA). Negative DNA extraction controls (lysis buffer and kit reagents only) were amplified and sequenced as contamination controls. The amplicons were normalized, pooled, and sequenced on the Illumina MiSeq platform using a V3 reagent kit with 2 × 300 cycles per sample and with imported and prepared routine data (samsheet) run in the MiSeq sequence program. After sequencing, Q30 scores were ≥70%, the percentage of clusters passing filter (i.e., cluster PF) was ≥80%, and there were at least 30,000 clean tags. Finally, image analysis and base calling were conducted with MiSeq Control Software.

### 2.5 Bioinformatic, predictive function, and statistical analyses

Based on the Quantitative Insights into Microbial Ecology bio‐informatic pipeline for performing taxonomy assignment by the operational taxonomic unit method, we used data of 113 sequences to analyze the fecal microbiota taxa. We further analyzed between‐class analysis by the principal component analysis (PCA) and clustering basis of the genus compositions (Arumugam et al., 2011), utilizing R statistics. We performed other analyses using statistically with SPSS version 19.0 (SPSS Inc., Chicago, Illinois), data were expressed as the mean ± standard deviation (*SD*). For continuous variables that conformed to a normal distribution, the difference between groups was analyzed by *t* test or one‐way ANOVA. The significant differences within groups were analyzed using ANOVA followed by post‐hoc Fisher's least significant difference (LSD) corrections for multiple comparisons when the analysis of variance was significant. For continuous variables that were not normally distributed, the difference between groups was analyzed using H test. We considered a two‐sided *p*‐value of < 0.05 to be statistically significant.

## 3 RESULTS

### 3.1 Patient characteristics and enterotype distribution

**Figure 1:**
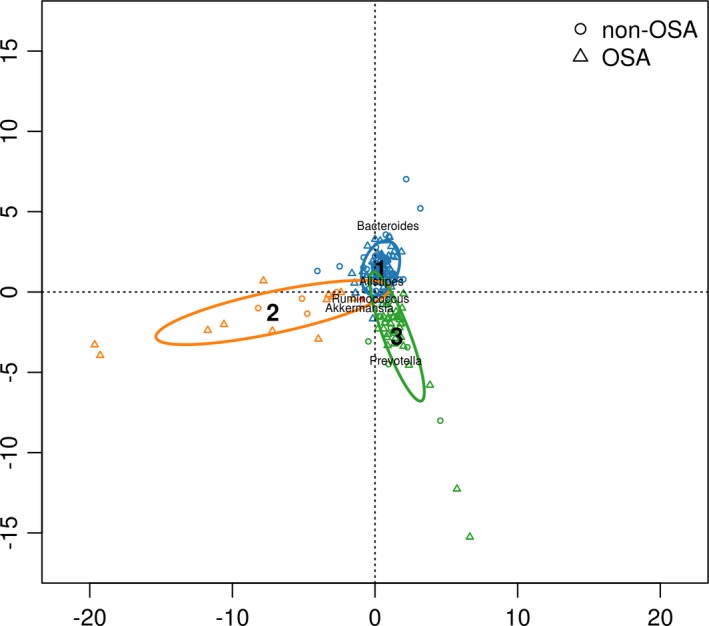
The fecal taxa class analysis in nonobstructive sleep apnoea–hypopnea syndrome (OSAHS) and OSAHS subjects of three enterotypes. Apnoea–hypopnea index (AHI) <15 as non‐OSA, AHI ≥ 15 as OSA. Enterotype 1: *Bacteroides* [non‐OSAHS (*n* = 41), OSAHS (*n* = 32)], Enterotype 2: *Ruminococcus* [non‐OSA (*n* = 6), OSAHS (*n* = 8)], Enterotype 3: *Prevotella* [non‐OSA (*n* = 14), OSAHS (*n* = 12)]

According to the PCA and clustering of fecal genus compositions for enterotypes class analysis, we enrolled 113 subjects [non‐OSAHS (*n* = 61), OSAHS (*n* = 52)] were divided according to three enterotypes: *Bacteroides* [non‐OSAHS (*n* = 41), OSAHS (*n* = 32)], *Ruminococcus* [non‐OSAHS (*n* = 6), OSAHS (*n* = 8)], and *Prevotella* [non‐OSAHS (*n* = 14), OSAHS (*n* = 12)] (Figure 1). Ages of *Ruminococcus* enterotype patients were significantly higher than those of the others enterotype patients (Table 1). BMI of *Prevotella* enterotype patients were significantly higher than *Bacteroides* enterotype patients (Table 1). The hip circumference of *Prevotella* enterotype patients were significantly higher than the others enterotype patients (Table 1).

**Table 1:** Participant characteristics

	*Bacteroides*	*Ruminococcus*	*Prevotella*	*F* value	*p* Value	post‐hoc test		
	enterotype	enterotype	enterotype			*p* Value		
	(*n* = 73)	(*n* = 14)	(*n* = 26)			*B* versus *R*	*B* versus *P*	*R* versus *P*
Gender (male/female)	61/12	11/3	20/6	NA^a^	NA	NA	NA	NA
Age (years, mean ± *SD*)	40.89 ± 10.56	53.14 ± 14.37	44.58 ± 13.51	6.560	0.002	<0.001	0.173	0.031
Height (cm)	166.28 ± 6.90	164.61 ± 7.30	166.83 ± 8.83	0.418	0.659	NA	NA	NA
Weight (kg)	72.51 ± 14.31	71.62 ± 14.25	79.41 ± 13.70	2.497	0.087	NA	NA	NA
Body mass index (kg m^−2^)	26.11 ± 3.87	26.44 ± 5.50	28.73 ± 5.27	3.382	0.038	0.799	0.011	0.122
Waist circumference (cm)	90.77 ± 9.87	91.21 ± 13.33	96.54 ± 12.27	2.742	0.069	NA	NA	NA
Hip circumference (cm)	96.95 ± 6.03	97.75 ± 10.42	103.21 ± 8.97	6.920	0.001	0.712	<0.001	0.028
Waist‐to‐hip ratio	0.94 ± 0.07	0.93 ± 0.06	0.93 ± 0.06	0.290	0.749	NA	NA	NA

Note

Values were calculated as mean ± *SD*.

a Not analyzed.

### 3.2 Cytokine analysis

**Figure 2:**
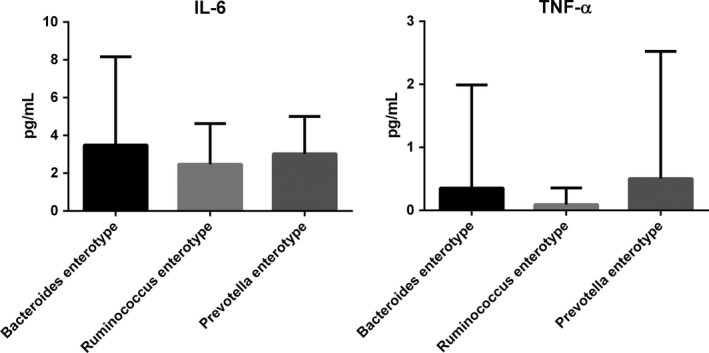
Cytokines levels analysis in three enterotypes subjects. IL: interleukin, TNF: tumor necrosis factor. Values were calculated as mean ± *SD*

There were not significantly different in IL‐ 6 and TNF‐α among three enterotypes patients (Figure 2).

### 3.3 PSG parameter analysis

Comparisons among patients with different enterotypes showed that total sleep arousal index was the lowest in the *Prevotella* enterotype patients (Table 2).

**Table 2:** Polysomnographic data analysis in three enterotypes subjects

	*Bacteroides*	*Ruminococcus*	*Prevotella*	F/*χ* ^2^ value	*p* value	post‐hoc test		
	enterotype	enterotype	enterotype			*p* value		
	(*n* = 73)	(*n* = 14)	(*n* = 26)			*B* versus *R*	*B* versus *P*	*R* versus *P*
Total sleep time (min)	633.42 ± 58.95	648.78 ± 51.83	641.11 ± 44.60	2.128	0.124	NA^a^	NA	NA
N1 sleep stage (min)	138.56 ± 66.92	152.04 ± 82.37	171.62 ± 96.22	1.819	0.167	NA	NA	NA
N1 sleep stage (%)	36.10 ± 15.90	42.19 ± 14.54	39.76 ± 17.28	1.122	0.329	NA	NA	NA
N2 sleep stage (min)	111.30 ± 60.67	85.85 ± 44.25	111.94 ± 57.67	3.052^b^	0.217^b^	NA	NA	NA
N2 sleep stage (%)	28.10 ± 11.99	24.45 ± 9.62	25.86 ± 10.48	0.814	0.446	NA	NA	NA
N3 sleep stage (min)	71.91 ± 36.85	57.71 ± 45.99	66.44 ± 41.66	0.839	0.435	NA	NA	NA
N3 sleep stage (%)	18.33 ± 8.29	15.95 ± 10.23	16.25 ± 9.79	0.790	0.456	NA	NA	NA
Non‐rapid eye movement (NREM) (min)	321.79 ± 87.88	295.60 ± 96.74	350.01 ± 98.5	1.740	0.180	NA	NA	NA
NREM (%)	82.56 ± 10.84	82.57 ± 7.62	81.87 ± 9.88	0.045	0.956	NA	NA	NA
Rapid eye movement (REM) (min)	67.21 ± 43.27	67.17 ± 37.26	79.63 ± 44.02	0.844	0.433	NA	NA	NA
REM (%)	17.43 ± 10.84	17.42 ± 7.62	18.12 ± 9.88	0.046	0.955	NA	NA	NA
Sleep efficiency (%)	70.40 ± 14.99	64.62 ± 21.45	75.92 ± 18.36	2.203	0.115	NA	NA	NA
Sleep latency	31.0 ± 29.96	31.7 ± 29.97	18.93 ± 20.36	4.726^b^	0.094^b^	NA	NA	NA
Wake after sleep onset	129.91 ± 71.69	174.76 ± 116.12	110.50 ± 98.33	2.643	0.076	NA	NA	NA
Arousal time (min)	157.64 ± 78.71	202.23 ± 131.03	128.14 ± 100.46	3.001	0.054	NA	NA	NA
Arousal times	20.35 ± 11.57	19.64 ± 12.70	14.50 ± 9.36	2.631	0.077	NA	NA	NA
Arousal index (events/h)	3.34 ± 2.06	3.86 ± 3.19	2.30 ± 1.68	3.087	0.050	0.414	0.036	0.031
Arousal time in REM	39.93 ± 38.94	39.42 ± 34.74	52.00 ± 36.13	3.870^b^	0.144^b^	NA	NA	NA
Arousal index in REM	32.25 ± 16.44	29.52 ± 17.66	38.96 ± 16.29	2.026	0.137	NA	NA	NA
Arousal time in NREM	210.15 ± 126.03	209.57 ± 148.79	288.19 ± 226.67	1.413^b^	0.493^b^	NA	NA	NA
Arousal index in NREM	38.17 ± 15.54	39.98 ± 16.20	45.76 ± 26.09	0.386^b^	0.824^b^	NA	NA	NA
Total sleep arousal times	250.08 ± 136.58	249.00 ± 166.24	340.19 ± 249.89	1.843^b^	0.398^b^	NA	NA	NA
Total sleep arousal index	38.06 ± 15.18	38.75 ± 15.83	44.64 ± 24.15	0.342^b^	0.843^b^	NA	NA	NA
Apnea‐hypopnea index (events/h)	19.63 ± 21.42	27.90 ± 22.63	27.41 ± 30.96	2.285^b^	0.319^b^	NA	NA	NA
Apnea‐hypopnea times	132.19 ± 170.66	180.50 ± 176.90	221.34 ± 278.37	1.627^b^	0.443^b^	NA	NA	NA
Obstructive apnea index (events/h)	8.85 ± 15.04	10.88 ± 12.44	10.62 ± 16.09	2.787^b^	0.248^b^	NA	NA	NA
Obstructive apnea times	62.78 ± 120.68	71.28 ± 102.99	85.26 ± 134.75	1.854^b^	0.396^b^	NA	NA	NA
Central apnea index (events/h)	0.46 ± 1.29	2.75 ± 8.64	1.92 ± 4.04	0.694^b^	0.707^b^	NA	NA	NA
Central apnea times	3.39 ± 9.45	11.78 ± 31.62	17.5 ± 40.21	0.778^b^	0.678^b^	NA	NA	NA
Mixed apnea index (events/h)	0.59 ± 1.52	1.61 ± 3.80	2.88 ± 6.79	1.664^b^	0.435^b^	NA	NA	NA
Mixed apnea times	4.56 ± 13.25	10.5 ± 27.95	25.73 ± 64.80	1.768^b^	0.413^b^	NA	NA	NA
Hypopnea index (events/h)	9.71 ± 8.95	12.63 ± 17.71	11.98 ± 14.29	0.036^b^	0.982^b^	NA	NA	NA
Hypopnea times	61.45 ± 55.31	86.92 ± 136.55	92.84 ± 125.09	0.388^b^	0.824^b^	NA	NA	NA
Longest apnea time (s)	37.80 ± 23.24	46.64 ± 27.65	45.26 ± 38.39	1.312^b^	0.519^b^	NA	NA	NA
Mean apnea‐hypopnea duration (s)	19.55 ± 8.98	20.25 ± 8.28	20.01 ± 9.78	0.049	0.952	NA	NA	NA
Longest hypopnea time (s)	68.26 ± 33.06	66.85 ± 27.29	78.50 ± 32.89	1.056	0.351	NA	NA	NA
Average hypopnea time (s)	28.3 ± 9.95	29.75 ± 12.62	31.52 ± 8.38	1.021	0.364	NA	NA	NA
Oxygen desaturation index (events/h)	18.72 ± 20.79	25.22 ± 23.56	25.9 ± 29.82	1.362^b^	0.506^b^	NA	NA	NA
Lowest oxygen saturation (%)	82.26 ± 8.81	79.35 ± 9.34	82.23 ± 10.88	0.584	0.559	NA	NA	NA
Average oxygen saturation (%)	94.34 ± 2.04	93.92 ± 3.34	94.15 ± 2.66	0.204	0.816	NA	NA	NA
Longest oxygen desaturation (s)	109.16 ± 42.98	95.17 ± 28.61	99.51 ± 40.54	1.011	0.367	NA	NA	NA
Mean heart rate	65.95 ± 10.32	65 ± 7.93	63.73 ± 9.55	0.495	0.611	NA	NA	NA
Arrhythmia index (events/h)	12.89 ± 54.36	38.04 ± 134.47	4.23 ± 8.19	1.428^b^	0.490^b^	NA	NA	NA
Maximum heart rate	99.41 ± 15.06	97.28 ± 12.19	96.92 ± 16.70	0.760^b^	0.684^b^	NA	NA	NA
Minimum heart rate	51.67 ± 8.97	53.28 ± 5.21	51.53 ± 6.68	0.255	0.775	NA	NA	NA
Blood pressure elevation index (events/h)	13.22 ± 15.21	16.43 ± 15.52	16.58 ± 22.43	0.757^b^	0.685^b^	NA	NA	NA
The highest systolic blood pressure (mmHg)	157.98 ± 39.08	148.57 ± 58.14	158.8 ± 45.51	0.308	0.736	NA	NA	NA
Average systolic blood pressure (mmHg)	120.41 ± 25.98	113.78 ± 42.26	118.19 ± 28.64	0.323	0.725	NA	NA	NA
Average diastolic blood pressure (mmHg)	81.80 ± 16.05	77.21 ± 24.46	82.92 ± 21.59	0.814^b^	0.666^b^	NA	NA	NA

Note

Values were calculated as mean ± *SD*.

a Not analyzed.

b Because the data do not meet the requirements of variance test, displayed as the Chi‐square test corresponding to the H test.

Using 15 as the AHI cutoff, when AHI ≥ 15, N1 sleep stage, arousal time in REM, and arousal index in REM were the highest in the *Prevotella* enterotype patients. Contrastingly, sleep latency and arousal time were the lowest in the *Prevotella* enterotype patients (Table 3).

**Table 3:** Polysomnographic data analysis in three enterotypes of apnoea–hypopnea index ≥ 15 patients

	*Bacteroides*	*Ruminococcus*	*Prevotella*	F/*χ* ^2^ value	*p* value	post‐hoc test		
	enterotype	enterotype	enterotype			*p* value		
	(*n* = 32)	(*n* = 8)	(*n* = 12)			*B* versus *R*	*B* versus *P*	*R* versus *P*
Total sleep time (min)	632.59 ± 71.61	663.63 ± 45.48	639.25 ± 41.52	1.803	0.176	NA^a^	NA	NA
N1 sleep stage (min)	162.46 ± 71.21	159.70 ± 98.14	236.23 ± 102.31	3.681	0.032	0.934	0.012	0.050
N1 sleep stage (%)	41.93 ± 15.78	41.18 ± 17.37	50.03 ± 16.75	1.199	0.310	NA	NA	NA
N2 sleep stage (min)	111.86 ± 65.07	95.24 ± 48.56	101.92 ± 56.09	0.292	0.748	NA	NA	NA
N2 sleep stage (%)	27.65 ± 11.46	24.23 ± 8.41	22.03 ± 9.76	1.308	0.280	NA	NA	NA
N3 sleep stage (min)	61.95 ± 35.56	63.06 ± 55.27	45.71 ± 37.88	0.805	0.453	NA	NA	NA
N3 sleep stage (%)	15.61 ± 7.96	16.54 ± 12.79	9.90 ± 7.97	2.101	0.133	NA	NA	NA
Non‐rapid eye movement (NREM) (min)	336.27 ± 92.94	318.00 ± 102.04	383.86 ± 111.29	1.355	0.267	NA	NA	NA
NREM (%)	85.20 ± 10.65	81.94 ± 5.83	81.96 ± 10.05	0.655	0.524	NA	NA	NA
Rapid eye movement (REM) (min)	56.65 ± 38.27	69.61 ± 33.06	76.83 ± 29.52	1.538	0.225	NA	NA	NA
REM (%)	14.80 ± 10.65	18.06 ± 5.83	18.04 ± 10.05	0.655	0.524	NA	NA	NA
Sleep efficiency (%)	70.68 ± 15.53	68.66 ± 19.05	81.52 ± 16.78	2.227	0.119	NA	NA	NA
Sleep latency	28.21 ± 26.64	26.26 ± 15.89	7.93 ± 5.48	12.911^b^	0.002^b^	0.703	0.001	0.005
Wake after sleep onset	131.92 ± 74.80	156.49 ± 119.11	77.79 ± 46.87	2.974	0.060	NA	NA	NA
Arousal time (min)	156.51 ± 76.52	177.55 ± 118.32	84.31 ± 48.77	4.547	0.015	0.504	0.009	0.013
Arousal times	19.34 ± 12.06	19.75 ± 14.53	12.50 ± 10.70	1.501	0.233	NA	NA	NA
Arousal index (events/h)	3.35 ± 2.52	3.89 ± 4.09	1.89 ± 1.83	1.713	0.191	NA	NA	NA
Arousal time in REM	34.81 ± 29.91	43.00 ± 34.32	66.67 ± 39.80	4.064	0.023	0.533	0.006	0.123
Arousal index in REM	33.37 ± 18.93	34.26 ± 17.70	50.00 ± 13.49	4.011	0.024	0.898	0.008	0.057
Arousal time in NREM	260.28 ± 152.59	262.13 ± 178.51	433.25 ± 262.42	4.190^b^	0.123^b^	NA	NA	NA
Arousal index in NREM	45.67 ± 17.02	46.20 ± 18.56	63.53 ± 28.16	3.491^b^	0.175^b^	NA	NA	NA
Total sleep arousal times	295.09 ± 160.4	305.13 ± 194.43	499.92 ± 285.72	4.389^b^	0.111^b^	NA	NA	NA
Total sleep arousal index	44.99 ± 16.94	44.41 ± 18.55	61.68 ± 25.41	3.919^b^	0.141^b^	NA	NA	NA
Apnea‐hypopnea index (events/h)	36.39 ± 22.86	42.04 ± 19.98	52.53 ± 29.78	3.884^b^	0.143^b^	NA	NA	NA
Apnea‐hypopnea times	247.09 ± 204.93	278.88 ± 176.82	430.58 ± 294.38	3.641^b^	0.162^b^	NA	NA	NA
Obstructive apnea index (events/h)	18.03 ± 19.14	14.43 ± 15.52	21.38 ± 18.74	0.560^b^	0.756^b^	NA	NA	NA
Obstructive apnea times	129.47 ± 159.54	99.88 ± 129.97	173.25 ± 159.52	1.534^b^	0.465^b^	NA	NA	NA
Central apnea index (events/h)	0.98 ± 1.83	4.81 ± 11.28	3.83 ± 5.40	0.731 b	0.694^b^	NA	NA	NA
Central apnea times	7.09 ± 13.44	20.63 ± 40.61	35.50 ± 54.65	0.578^b^	0.749^b^	NA	NA	NA
Mixed apnea index (events/h)	1.31 ± 2.10	2.78 ± 4.81	6.19 ± 9.08	1.852^b^	0.396^b^	NA	NA	NA
Mixed apnea times	10.13 ± 18.71	18.13 ± 36.00	55.33 ± 88.13	2.091^b^	0.351^b^	NA	NA	NA
Hypopnea index (events/h)	16.08 ± 9.56	19.99 ± 20.79	21.17 ± 16.88	0.734	0.485	NA	NA	NA
Hypopnea times	100.41 ± 57.37	140.25 ± 163.59	166.50 ± 155.08	0.977^b^	0.614^b^	NA	NA	NA
Longest apnea time (s)	50.31 ± 19.59	47.63 ± 29.34	69.08 ± 38.55	2.494	0.093	NA	NA	NA
Mean apnea‐hypopnea duration (s)	23.20 ± 6.35	20.30 ± 6.90	26.97 ± 7.53	2.531	0.090	NA	NA	NA
Longest hypopnea time (s)	82.69 ± 28.53	70.25 ± 25.97	99.75 ± 24.15	3.044	0.057	NA	NA	NA
Average hypopnea time (s)	31.29 ± 6.58	28.53 ± 6.55	35.74 ± 8.24	2.871	0.066	NA	NA	NA
Oxygen desaturation index (events/h)	34.23 ± 22.94	38.98 ± 22.58	49.73 ± 29.24	1.758	0.183	NA	NA	NA
Lowest oxygen saturation (%)	76.31 ± 9.20	76.00 ± 10.01	74.83 ± 12.04	0.095	0.909	NA	NA	NA
Average oxygen saturation (%)	93.31 ± 2.32	93.13 ± 4.09	92.50 ± 2.81	1.111^b^	0.574^b^	NA	NA	NA
Longest oxygen desaturation (s)	113.18 ± 37.43	103.39 ± 23.54	92.93 ± 26.57	1.646	0.203	NA	NA	NA
Mean heart rate	66.56 ± 10.38	67.25 ± 6.67	67.83 ± 10.83	0.074	0.929	NA	NA	NA
Arrhythmia index (events/h)	5.29 ± 13.97	65.06 ± 177.85	3.82 ± 5.03	0.784^b^	0.676^b^	NA	NA	NA
Maximum heart rate	99.09 ± 14.79	99.75 ± 15.64	103.08 ± 18.70	0.278	0.758	NA	NA	NA
Minimum heart rate	51.88 ± 7.07	54.50 ± 4.44	53.50 ± 6.56	0.626	0.539	NA	NA	NA
Blood pressure elevation index (events/h)	20.91 ± 18.96	20.44 ± 18.45	26.09 ± 27.79	0.288	0.751	NA	NA	NA
The highest systolic blood pressure (mmHg)	172.97 ± 35.54	180.75 ± 38.26	163.25 ± 62.49	0.414	0.663	NA	NA	NA
Average systolic blood pressure (mmHg)	130.44 ± 22.64	138.50 ± 22.91	117.58 ± 41.10	1.508	0.231	NA	NA	NA
Average diastolic blood pressure (mmHg)	87.28 ± 13.38	88.00 ± 9.94	84.25 ± 29.45	0.150	0.861	NA	NA	NA

Note

Values were calculated as mean ± *SD*.

a Not analyzed.

b Because the data do not meet the requirements of variance test, displayed as the Chi‐square test corresponding to the H test.

For *Prevotella* enterotype patients, N1 sleep stage, N3 sleep stage, arousal index in REM, arousal time in NREM, arousal index in NREM, total sleep arousal times, total sleep arousal index, AHI, apnea‐hypopnea times, obstructive apnea index, obstructive apnea times, central apnea index, mixed apnea index, hypopnea index, hypopnea times, longest apnea time, mean apnea‐hypopnea duration (MAD), longest hypopnea time, average hypopnea time, oxygen desaturation index, and mean heart rate were significantly elevated in AHI ≥ 15 patients. However, sleep latency, arousal time, lowest oxygen saturation, and average oxygen saturation were significantly decreased in AHI ≥ 15 patients (Table 4).

**Table 4:** Polysomnographic data analysis in *Prevotella* enterotype subjects

	AHI ＜15	AHI ≥ 15
Total sleep time (min)	403.04 ± 118.21	460.69 ± 117.35
N1 sleep stage (min)	**116.25 ± 42.34**	**236.23 ± 102.31** **
N1 sleep stage (%)	**30.96 ± 12.49**	**50.03 ± 16.75** **
N2 sleep stage (min)	120.54 ± 59.68	101.92 ± 56.09
N2 sleep stage (%)	29.15 ± 10.26	22.03 ± 9.76
N3 sleep stage (min)	**84.21 ± 37.23**	**45.71 ± 37.88** *
N3 sleep stage (%)	**21.70 ± 7.83**	**9.90 ± 7.97** **
Non‐rapid eye movement (NREM) (min)	321.00 ± 78.85	383.86 ± 111.29
NREM (%)	81.80 ± 10.11	81.96 ± 10.05
Rapid eye movement (REM) (min)	82.04 ± 54.54	76.83 ± 29.52
REM (%)	18.20 ± 10.11	18.04 ± 10.05
Sleep efficiency (%)	71.13 ± 18.87	81.52 ± 16.78
Sleep latency	**28.36 ± 23.75**	**7.93 ± 5.48** **
Wake after sleep onset	138.54 ± 122.07	77.79 ± 46.87
Arousal time (min)	**165.71 ± 118.75**	**84.31 ± 48.77** *
Arousal times	16.21 ± 8.06	12.50 ± 10.70
Arousal index (events/h)	2.66 ± 1.52	1.89 ± 1.83
Arousal time in REM	39.43 ± 28.30	66.67 ± 39.80
Arousal index in REM	**29.50 ± 12.13**	**50.00 ± 13.49** ***
Arousal time in NREM	**163.86 ± 66.82**	**433.25 ± 262.42** **
Arousal index in NREM	**30.54 ± 9.82**	**63.53 ± 28.16** **
Total sleep arousal times	**203.29 ± 85.31**	**499.92 ± 285.72** **
Total sleep arousal index	**30.04 ± 8.81**	**61.68 ± 25.41** **
Apnea‐hypopnea index (events/h)	**5.88 ± 3.43**	**52.53 ± 29.78** ***
Apnea‐hypopnea times	**42.00 ± 25.31**	**430.58 ± 294.38** **
Obstructive apnea index (events/h)	**1.41 ± 1.65**	**21.38 ± 18.74** **
Obstructive apnea times	**9.86 ± 10.79**	**173.25 ± 159.52** **
Central apnea index (events/h)	**0.30 ± 0.77**	**3.83 ± 5.40** *
Central apnea times	2.07 ± 5.14	35.50 ± 54.65
Mixed apnea index (events/h)	**0.05 ± 0.09**	**6.19 ± 9.08** *
Mixed apnea times	0.36 ± 0.63	55.33 ± 88.13
Hypopnea index (events/h)	**4.11 ± 2.65**	**21.17 ± 16.88** **
Hypopnea times	**29.71 ± 21.00**	**166.50 ± 155.08** **
Longest apnea time (s)	**24.86 ± 24.59**	**69.08 ± 38.55** **
Mean apnea‐hypopnea duration (s)	**14.06 ± 7.29**	**26.97 ± 7.53** ***
Longest hypopnea time (s)	**60.29 ± 28.51**	**99.75 ± 24.15** **
Average hypopnea time (s)	**27.91 ± 6.86**	**35.74 ± 8.24** *
Oxygen desaturation index (events/h)	**5.48 ± 3.66**	**49.73 ± 29.24** ***
Lowest oxygen saturation (%)	**88.57 ± 3.34**	**74.83 ± 12.04** ***
Average oxygen saturation (%)	**95.00 ± 1.79**	**92.50 ± 2.81** **
Longest oxygen desaturation (s)	84.23 ± 33.17	92.93 ± 26.57
Mean heart rate	**62.00 ± 9.08**	**67.83 ± 10.83** *
Arrhythmia index (events/h)	2.02 ± 2.09	3.82 ± 5.03
Maximum heart rate	94.00 ± 4.60	103.08 ± 18.70
Minimum heart rate	51.67 ± 6.12	53.50 ± 6.56
Blood pressure elevation index (events/h)	11.10 ± 9.49	26.09 ± 27.79
The highest systolic blood pressure (mmHg)	105.67 ± 53.60	163.25 ± 62.49
Average systolic blood pressure (mmHg)	80.83 ± 40.36	117.58 ± 41.10
Average diastolic blood pressure (mmHg)	62.83 ± 31.35	84.25 ± 29.45

Note

Values were calculated as mean ± *SD*.

AHI: apnoea–hypopnea index.

* *p* < 0.05,

** *p* < 0.01,

*** *p* < 0.001 compared with AHI < 15 subjects.

## 4 DISCUSSION

OSAHS is a systemic and comprehensive disorder associated with comorbidities, including cardiovascular diseases and metabolic abnormalities. Thus, the IH mechanism alone is insufficient to interpret the complete pathogenesis of OSAHS because OSAHS is also affected by several other aspects, including the CNS causing neuropsychiatric and neurodegenerative disorders (Farré et al., 2018; Gaspar et al., 2017; Lavie, 2015; Rosenzweig et al., 2014). This study shows that arousal‐related parameters or sleep stages are significantly disrupted in AHI ≥ 15 patients with *Prevotella* enterotypes; further analysis, obstructive, central, and mixed apnea indices, and mean heart rate are also significantly elevated in AHI ≥ 15 patients.

*Prevotella* enterotype is associated with diets high in carbohydrates (fiber) and simple sugars. *Bacteroides* enterotype is associated with Western‐style diets, including consuming high amounts of protein and fat, whereas *Ruminococcus* species enterotype is linked to nondigestible carbohydrates (Conlon & Bird, 2015). Despite the fact that the *Bacteroides* predominant enterotype seems to be more common in IBD patients, the *Prevotella* enterotype is more representative in healthy subjects (Costea et al., 2018; Knights et al., 2014). Our findings show that *Bacteroides* enterotype patients are not susceptible to OSAHS, in contrast to the susceptibility of *Prevotella* enterotype patients.

IH‐exposed mice mimic OSAHS, causing profound alterations in gut microbiota. Hypoxia/re‐oxygenation is the most pronounced (Moreno‐Indias et al., 2015), inducing an alteration in intestinal epithelial barrier markers and increasing intestinal permeability, leading to local and systemic inflammatory responses and consequent multi‐organ morbidities (Barceló et al., 2016; Grootjans et al., 2010). However, only *Bacteroides* and *Prevotella* enterotypes can be classified in the rodent model, IH‐exposed mice classify as the *Prevotella* enterotype (Moreno‐Indias et al., 2015), which is similar to our particularly OSAHS patients. It has further been shown that IH leads to gut microbiota alteration and accompanying endotoxin production (Maes, Kubera, & Leunis, 2008). It is that IH model creates an anoxic environment in the intestine, which is beneficial for obligate anerobic bacterial growth, endogenous LPS production from gram‐negative bacteria, and triggering inflammation. Notably, *Prevotella* is a genus of gram‐negative anerobic bacteria, and it has a tendency to alter intestinal permeability (Moreno‐Indias et al., 2015, 2016). Although this evidence only reveals the IH contribution to the pathogenesis, we also speculated that SF is another principal contributor (Poroyko et al., 2016). SF‐induced mice manifest inflammation and enhanced production of endotoxins produced by gut microbiota, too (Poroyko et al., 2016). Taken together, LPS may play a crucial role in driving systemic inflammation, it has been shown in IH and SF modeling OSAHS models (Moreno‐Indias et al., 2015, 2016; Poroyko et al., 2016). Moreover, in middle‐aged non‐obese males with OSAHS, disruption of the intestinal barrier, and concurrent increased serum d‐lactate levels possibly contribute to intestinal hyperpermeability and are significantly positively associated with pro‐inflammatory IL‐1β, IL‐6, and TNF‐α cytokines in serum (Heizati et al., 2017) in which TNF‐α elevation in *Prevotella* enterotype subjects is similar with our results, but it did not reach statistically significant differences.

*Prevotella* enterotype patients with AHI ≥ 15 in our results, suggesting that LPS production triggers downstream signaling pathways, leading to the subsequent release of pro‐inflammatory cytokines (Tobias, Soldau, & Ulevitch, 1989). Furthermore, the elevation of LPS‐binding protein is also verified in OSAHS mimicking rodent models (Poroyko et al., 2016) and patients (Heizati et al., 2017), particularly regarding in the higher d‐lactate level of OSAHS patients. Inflammatory mediators can be produced by peripheral and central cells. Peripheral inflammatory mediators may invade the CNS by crossing the blood–brain barrier, affecting behaviors and causing metabolic problems and psychiatric disorders (Ko & Liu, 2016). Here, our data suggest that the gut microbiota might impact the brain in OSAHS patients by modulating inflammatory responses.

Additionally, we should mention that the *Prevotella* enterotype is linked to diets rich in simple sugars. Simple carbohydrate consumption has been hypothesized to be related to elevated BP values and obesity (Orlando, Cazzaniga, Giussani, Palestini, & Genovesi, 2018), as showed in our data. *Prevotella* enterotype patients had a higher BMI and hip circumference than *Bacteriodes* enterotype patients. Monosaccharide intake induces inflammation in epithelial cells and contributes to hypertension (Orlando et al., 2018), linking to LPS production, which can stimulate systemic inflammatory cascades (Moreno‐Indias et al., 2015). Inflammation mediates the pathogenesis of many physiological dysfunctions, such as metabolic syndrome and mental dysfunction (Ko & Liu, 2016), and thus might ultimately result in OSAHS‐related metabolic comorbidities. Although *Ruminococcus* is associated with resistant starch, host health benefits from short chain fatty acids that have been demonstrated to regulate immune inflammatory responses (Macfarlane & Macfarlane, 2012). The enriched bacteria *Ruminococcus* spp. and *Sutterella* spp. are found in psychiatric patients (Wang et al., 2013). The abovementioned literature supports the hypothesis that microbiota disruption influences the pathophysiological process of OSAHS might be through a microbiota–gut–brain axis.

Despite OSAHS is one of the most common sleep apnea syndromes (SAS), other types are mixed sleep apnea (MSA) and central sleep apnea (CSA). The prevalence of OSAHS, complex SAS (CompSAS), and central SAS are 84.0%, 15.0%, and 0.4%, respectively (Morgenthaler, Kagramanov, Hanak, & Decker, 2006). MSA generally describes the mixture of both obstructive and central apnea events during diagnostic sleep, although many central apnea indices occurrence is also identified as MSA, which is sometimes referred to as CompSAS (Khan & Franco, 2014). Whereas CompSAS is a form of CAS wherein the persistence or emergence of central apneas or hypopneas has disappeared with CPAP, patients have predominately obstructive or mixed apneas occurring at ≥ 5 events/h (Gay, 2008). Additionally, reportedly, there is a high prevalence of hypertension and heart disease in patients with CompSAS (Westhoff, Arzt, & Litterst, 2012). In our data, all of apnea indices were significantly increased in *Prevotella* enterotype patients with AHI ≥ 15. Thus, abnormalities in electrocardiography, electroencephalography, electromyography, and electro‐oculography results should be more concern.

Both IH and SF have been demonstrated to independently affect similar CNS regions in animal research (Rosenzweig et al., 2014). The N1 sleep stage is associated with the transition from wakefulness to other sleep stages or the following arousal during sleep. A higher N1 percentage might mean more events of wakefulness and/or arousal, SF (episodic arousal from sleep), and sympathetic overactivity during sleep (Shao et al., 2018). REM sleep dysregulation significantly contributes to cognitive distortions and dysfunctions that rely on emotion and memory functions are also affected (Palagini et al., 2013). Moreover, the effects of sleep deprivation on cognition have been investigated (Killgore, 2010). Thus, OSAHS patients have been found to have neurocognitive and emotional disorders, suggesting the modulation of various neurotransmitters during the sleep period (Rosenzweig et al., 2014). Recently, a multicenter randomized controlled trial has been initiated evaluating the extent to which CPAP treatment improves neurocognitive dysfunction in OSAHS patients and examining the role of gut microbiota in this change (Xu et al., 2017). Preliminary results suggest the viability of the hypothesis that microbiota modulate central nervous functions in OSAHS patients.

Although the neural mechanisms underlying SAS‐induced brain injury have not been completely elucidated, repeated arousals enable the characterization of the different stages of sleep. In this study, the N1 sleep stage, MAD, and arousal index were increased in *Prevotella* enterotype patients. BP was not significantly different among the three enterotype AHI ≥ 15 patient groups, but mean diastolic pressure during sleep was > 80 mmHg, which was similar to that observed in a previous study (Shao et al., 2018). MAD can act as an indicator of the levels of sleep parameters and blood oxygenation for the evaluation of severe OSAHS patients (Zhan, Fang, Wu, Pinto, & Wei, 2018). When MAD is elevated, sleep apnea appears to be more likely to cause respiratory arousal and might impair sleep stability, resulting in SF. This outcome might then be that the transition of the N2 sleep stage (the longest stage of sleep) to the N3 sleep stage is a vulnerable period, which is interrupted in OSAHS patients, and the overall sleep pattern becomes light sleep (Zhan et al., 2018). Additionally, chronic SF induction elevates fat mass, alters fecal microbiota, promotes increased gut permeability, leads to systemic and adipose tissue inflammatory changes, and accompanies metabolic dysfunction (Poroyko et al., 2016). These symptoms are known to be associated with OSAHS‐related metabolic comorbidities, implying that the microbiota–gut–brain axis has a biaxial effect on the development of OSAHS pathology.

Contrastingly, evidence has shown that N1, N3, and REM sleep stages decrease and the N2 sleep stage increases in OSAHS patients (Rosenzweig et al., 2014). However, a higher N1 percentage, a longer MAD, and a shortened REM sleep stage were revealed in AHI ≥ 15 patients with OSAHS‐induced hypertension (Shao et al., 2018; Zhan et al., 2018). Our findings reveal that BP plays a vital role, particularly for SAS, where BP is comprehensively regulated by the peripheral and central systems. Hence, future studies should re‐examine these questions in subgroups of hypertensive and normotensive OSAHS patients to evaluate their general applicability.

In summary, this study initiates a new approach to the study of sleep apnea through a combination of polysomnographic measurements with analysis of enterotypes. Obstructive, central, and mixed apnea indices, N1 and N3 sleep stages, MAD, arousal indices, and mean heart rate were all prominently increased in AHI ≥ 15 patients with the *Prevotella* enterotype. Our results raise the possible association that the microbiota–gut–brain axis operates bidirectionally, with significant impact on the pathogenesis of OSAHS including functions of the gut and brain that eventually contribute to multiple end‐organ morbidities.

## CONTRIBUTORS

Conceptualization: C.Y.K., H.P.Z., and Y.M.Z.; Investigation: C.Y.K., A.K.H., J.M.F., L.M.H., J.H.Y., and H.Z.S.; Data curation and Visualization: C.Y.K., A.K.H., J.M.F., F.R.Y., H.P.Z., and Y.M.Z.; Writing‐Review and Editing: C.Y.K., H.P.Z., and Y.M.Z.

## DECLARATIONS OF CONFLICT OF INTEREST

The authors declare that they have no financial and personal relationships with others that may inappropriately influence the results and interpretation in this manuscript.

## References

[brb31287-bib-0001] Arumugam, M. , Raes, J. , Pelletier, E. , Le Paslier, D. , Yamada, T. , Mende, D. R. , … Bork, P. (2011). Enterotypes of the human gut microbiome. Nature, 473(7346), 174–180. https://doi.10.1038/nature09944 21508958 10.1038/nature09944 PMC3728647

[brb31287-bib-0002] Barceló, A. , Esquinas, C. , Robles, J. , Piérola, J. , De la Peña, M. , Aguilar, I. , … Barbé, F. (2016). Gut epithelial barrier markers in patients with obstructive sleep apnea. Sleep Medicine, 26, 12–15. https://doi.10.1016/j.sleep.2016.01.019 28007354 10.1016/j.sleep.2016.01.019

[brb31287-bib-0003] Conlon, M. A. , & Bird, A. R. (2015). The impact of diet and lifestyle on gut microbiota and human health. Nutrients, 7(1), 17–44. https://doi.10.3390/nu7010017 10.3390/nu7010017 PMC4303825 25545101

[brb31287-bib-0004] Costea, P. I. , Hildebrand, F. , Arumugam, M. , Bäckhed, F. , Blaser, M. J. , Bushman, F. D. , … Bork, P. (2018). Enterotypes in the landscape of gut microbial community composition. Nature Microbiology, 3(1), 8–16. https://doi.10.1038/s41564‐017‐0072‐8 10.1038/s41564-017-0072-8 PMC5832044 29255284

[brb31287-bib-0005] Farré, N. , Farré, R. , & Gozal, D. (2018). Sleep apnea morbidity: A consequence of microbial‐immune cross‐talk? Chest, 154(4), 754–759. https://doi.10.1016/j.chest.2018.03.001 29548630 10.1016/j.chest.2018.03.001

[brb31287-bib-0006] Gaspar, L. S. , Álvaro, A. R. , Moita, J. , & Cavadas, C. (2017). Obstructive sleep apnea and hallmarks of aging. Trends in Molecular Medicine, 23(8), 675–692. 10.1016/j.molmed.2017.06.006 28739207

[brb31287-bib-0007] Gay, P. C. (2008). Complex sleep apnea: It really is a disease. Journal of Clinical Sleep Medicine, 4(5), 403–405 18853694 PMC2576323

[brb31287-bib-0008] Grootjans, J. , Thuijls, G. , Verdam, F. , Derikx, J. P. , Lenaerts, K. , & Buurman, W. A. (2010). Non‐invasive assessment of barrier integrity and function of the human gut. World Journal of Gastrointestinal Surgery, 2, 61–69. https://doi.10.4240/wjgs.v2.i3.61 21160852 10.4240/wjgs.v2.i3.61 PMC2999221

[brb31287-bib-0009] Heizati, M. , Li, N. , Shao, L. , Yao, X. , Wang, Y. , Hong, J. , … Abulikemu, S. (2017). Does increased serum d‐lactate mean subclinical hyperpermeability of intestinal barrier in middle‐aged nonobese males with OSA? Medicine, 96(49), e9144. https://doi.10.1097/MD.0000000000009144 29245360 10.1097/MD.0000000000009144 PMC5728975

[brb31287-bib-0010] Kao, Y. C. , Ko, C. Y. , Wang, S. C. , & Liu, Y. P. (2016). Protective effects of quetiapine on metabolic and inflammatory abnormalities in schizophrenic patients during exacerbated stage. The Chinese Journal of Physiology, 59(2), 69–77. https://doi.10.4077/CJP.2016.BAE370 27080462 10.4077/CJP.2016.BAE370

[brb31287-bib-0011] Khan, M. T. , & Franco, R. A. (2014). Complex sleep apnea syndrome. Sleep Disorders, 2014, 798487. https://doi.10.1155/2014/798487 24693440 10.1155/2014/798487 PMC3945285

[brb31287-bib-0012] Killgore, W. D. (2010). Effects of sleep deprivation on cognition. Progress in Brain Research, 185, 105–129. https://doi.10.1016/B978‐0‐444‐53702‐7.00007‐5 21075236 10.1016/B978-0-444-53702-7.00007-5

[brb31287-bib-0013] Knights, D. , Ward, T. L. , McKinlay, C. E. , Miller, H. , Gonzalez, A. , McDonald, D. , & Knight, R. (2014). Rethinking "enterotypes". Cell Host & Microbe, 16(4), 433–437. https://doi.10.1016/j.chom.2014.09.013 25299329 10.1016/j.chom.2014.09.013 PMC5558460

[brb31287-bib-0014] Ko, C. Y. , & Liu, Y. P. (2016). Disruptions of sensorimotor gating, cytokines, glycemia, monoamines, and genes in both sexes of rats reared in social isolation can be ameliorated by oral chronic quetiapine administration. Brain, Behavior, and Immunity, 51, 119–130. https://doi.10.1016/j.bbi.2015.08.003 10.1016/j.bbi.2015.08.003 26254231

[brb31287-bib-0015] Lavie, L. (2015). Oxidative stress in obstructive sleep apnea and intermittent hypoxia‐revisited‐the bad ugly and good: Implications to the heart and brain. Sleep Medicine Reviews, 20, 27–45. https://doi.10.1016/j.smrv.2014.07.003 25155182 10.1016/j.smrv.2014.07.003

[brb31287-bib-0016] Macfarlane, G. T. , & Macfarlane, S. (2012). Bacteria, colonic fermentation, and gastrointestinal health. Journal of AOAC International, 95(1), 50–60. https://doi.10.5740/jaoacint.SGE_Macfarlane 22468341 10.5740/jaoacint.sge_macfarlane

[brb31287-bib-0017] Maes, M. , Kubera, M. , & Leunis, J. C. (2008). The gut‐brain barrier in major depression: Intestinal mucosal dysfunction with an increased translocation of LPS from gram negative enterobacteria (leaky gut) plays a role in the inflammatory pathophysiology of depression. Neuroendocrinology Letters, 29(1), 117–124. https://doi.10.1038/ncpendmet0726 18283240

[brb31287-bib-0018] Moreno‐Indias, I. , Torres, M. , Montserrat, J. M. , Sanchez‐Alcoholado, L. , Cardona, F. , Tinahones, F. J. , … Farré, R. (2015). Intermittent hypoxia alters gut microbiota diversity in a mouse model of sleep apnoea. European Respiratory Journal, 45(4), 1055–1065. https://doi.10.1183/09031936.00184314 25537565 10.1183/09031936.00184314

[brb31287-bib-0019] Moreno‐Indias, I. , Torres, M. , Sanchez‐Alcoholado, L. , Cardona, F. , Almendros, I. , Gozal, D. , … Farré, R. (2016). Normoxic recovery mimicking treatment of sleep apnea does not reverse intermittent hypoxia‐induced bacterial dysbiosis and low‐grade endotoxemia in mice. Sleep, 39(10), 1891–1897. https://doi.10.5665/sleep.6176 27397563 10.5665/sleep.6176 PMC5020371

[brb31287-bib-0020] Morgenthaler, T. I. , Kagramanov, V. , Hanak, V. , & Decker, P. A. (2006). Complex sleep apnea syndrome: Is it a unique clinical syndrome? Sleep, 29, 1203–1209. https://doi.10.1093/sleep/29.9.1203 17040008 10.1093/sleep/29.9.1203

[brb31287-bib-0021] Orlando, A. , Cazzaniga, E. , Giussani, M. , Palestini, P. , & Genovesi, S. (2018). Hypertension in children: Role of obesity, simple carbohydrates, and uric acid. Frontiers in Public Health, 6, 129. https://doi.10.3389/fpubh.2018.00129 29774210 10.3389/fpubh.2018.00129 PMC5943632

[brb31287-bib-0022] Palagini, L. , Baglioni, C. , Ciapparelli, A. , Gemignani, A. , & Riemann, D. (2013). REM sleep dysregulation in depression: State of the art. Sleep Medicine Reviews, 17(5), 377–390. https://doi.10.1016/j.smrv.2012.11.001 23391633 10.1016/j.smrv.2012.11.001

[brb31287-bib-0023] Poroyko, V. A. , Carreras, A. , Khalyfa, A. , Khalyfa, A. A. , Leone, V. , Peris, E. , … Gozal, D. (2016). Chronic sleep disruption alters gut microbiota, induces systemic and adipose tissue inflammation and insulin resistance in mice. Scientific Reports, 6, 35405. https://doi.10.1038/srep35405 27739530 10.1038/srep35405 PMC5064361

[brb31287-bib-0024] Rosenzweig, I. , Williams, S. C. , & Morrell, M. J. (2013). Cross Talk opposing view: The intermittent hypoxia attending severe obstructive sleep apnoea does not lead to alterations in brain structure and function. The Journal of Physiology, 591(2), 383–385. https://doi.10.1113/jphysiol.2012.241224 23322287 10.1113/jphysiol.2012.241224 PMC3577530

[brb31287-bib-0025] Rosenzweig, I. , Williams, S. C. , & Morrell, M. J. (2014). The impact of sleep and hypoxia on the brain: Potential mechanisms for the effects of obstructive sleep apnea. Current Opinion in Pulmonary Medicine, 20(6), 565–571. https://doi.10.1097/MCP.0000000000000099 25188719 10.1097/MCP.0000000000000099

[brb31287-bib-0026] Shao, L. , Heizhati, M. , Yao, X. , Wang, Y. , Abulikemu, S. , Zhang, D. , … Li, N. (2018). Influences of obstructive sleep apnea on blood pressure variability might not be limited only nocturnally in middle‐aged hypertensive males. Sleep Breath, 22(2), 377–384. https://doi.10.1007/s11325‐017‐1571‐9 29150775 10.1007/s11325-017-1571-9

[brb31287-bib-0027] Sherwin, E. , Sandhu, K. V. , Dinan, T. G. , & Cryan, J. F. (2016). May the force be with you: The light and dark sides of the microbiota‐gut‐brain axis in neuropsychiatry. CNS Drugs, 30(11), 1019–1041. https://doi.10.1007/s40263‐016‐0370‐3 27417321 10.1007/s40263-016-0370-3 PMC5078156

[brb31287-bib-0028] Singh, R. K. , Chang, H.‐W. , Yan, D. i. , Lee, K. M. , Ucmak, D. , Wong, K. , … Liao, W. (2017). Influence of diet on the gut microbiome and implications for human health. Journal of Translational Medicine, 15(1), 73. https://doi.10.1186/s12967‐017‐1175‐y 28388917 10.1186/s12967-017-1175-y PMC5385025

[brb31287-bib-0029] Tobias, P. S. , Soldau, K. , & Ulevitch, R. J. (1989). Identification of a lipid A binding site in the acute phase reactant lipopolysaccharide binding protein. Journal of Biological Chemistry, 264(18), 10867–10871 2471708

[brb31287-bib-0030] Wang, L. , Christophersen, C. T. , Sorich, M. J. , Gerber, J. P. , Angley, M. T. , & Conlon, M. A. (2013). Increased abundance of *Sutterella* spp. and *Ruminococcus* torques in feces of children with autism spectrum disorder. Molecular Autism, 4(1), 42. https://doi.10.1186/2040-2392-4-42 24188502 10.1186/2040-2392-4-42 PMC3828002

[brb31287-bib-0031] Westhoff, M. , Arzt, M. , & Litterst, P. (2012). Prevalence and treatment of central sleep apnoea emerging after initiation of continuous positive airway pressure in patients with obstructive sleep apnoea without evidence of heart failure. Sleep Breath, 16(1), 71–78. https://doi.10.1007/s11325‐011‐0486‐0 21347650 10.1007/s11325-011-0486-0

[brb31287-bib-0032] Xu, H. , Wang, H. , Guan, J. , Yi, H. , Qian, Y. , Zou, J. , … Yin, S. (2017). Effects of continuous positive airway pressure on neurocognitive architecture and function in patients with obstructive sleep apnoea: Study protocol for a multicentre randomised controlled trial. British Medical Journal Open, 7(5), e014932. https://doi.10.1136/bmjopen‐2016‐014932 10.1136/bmjopen-2016-014932 PMC5729992 28550021

[brb31287-bib-0033] Zhan, X. , Fang, F. , Wu, C. , Pinto, J. M. , & Wei, Y. (2018). A retrospective study to compare the use of the mean apnea‐hypopnea duration and the apnea‐hypopnea index with blood oxygenation and sleep patterns in patients with obstructive sleep apnea diagnosed by polysomnography. Medical Science Monitor, 4, 1887–1893. https://doi.10.12659/MSM.909219 10.12659/MSM.909219 PMC5892846 29603712

